# A Multidisciplinary survey on controversies in the use of EUS-guided FNA: assessing perspectives of surgeons, oncologists and gastroenterologists

**DOI:** 10.1186/1471-230X-11-117

**Published:** 2011-11-02

**Authors:** Jesse Lachter, Yoav Rosenthal, Yoram Kluger

**Affiliations:** 1 Department of Gastroenterology, Rambam Health Care Campus, Bat Galim, Haifa, Israel; 2 The Rappaport Faculty of Medicine, Technion- Israel Institute of Technology, Efron St, Bat Galim, Haifa, Israel; 3 Department of Surgery, Rambam Health Care Campus, Bat Galim, Haifa, Israel

## Abstract

**Background:**

EUS-guided FNA can help diagnose and differentiate between various pancreatic and other lesions.

The aim of this study was to compare approaches among involved/relevant physicians to the controversies surrounding the use of FNA in EUS.

**Methods:**

A five-case survey was developed, piloted, and validated. It was collected from a total of 101 physicians, who were all either gastroenterologists (GIs), surgeons or oncologists. The survey compared the management strategies chosen by members of these relevant disciplines regarding EUS-guided FNA.

**Results:**

For CT operable T2NOM0 pancreatic tumors the research demonstrated variance as to whether to undertake EUS-guided FNA, at p < 0.05. For inoperable pancreatic tumors 66.7% of oncologists, 62.2% of surgeons and 79.1% of GIs opted for FNA (p < 0.05). For cystic pancreatic lesions, oncologists were more likely to send patients to surgery without FNA. For stable simple pancreatic cysts (23 mm), most physicians (66.67%) did not recommend FNA. For a submucosal gastric 19 mm lesion, 63.2% of surgeons recommended FNA, vs. 90.0% of oncologists (p < 0.05).

**Conclusions:**

Controversies as to ideal application of EUS-FNA persist. Optimal guidelines should reflect the needs and concerns of the multidisciplinary team who treat patients who need EUS-FNA. Multi-specialty meetings assembled to manage patients with these disorders may be enlightening and may help develop consensus.

## Background

Pancreatic cancer (PCA) is the fourth leading cause of cancer-related death in the United States [1]. Although relatively common, it is considered challengingly difficult both to diagnose early and to treat. Pancreatic solid tumors are often malignant, with adenocarcinoma being the most prevalent histological form. However, about 10% of the tumors are of various other histologies, with different natural history and management considerations [2]. Currently, the only option for a cure for PCA patients begins with surgical extirpation of the tumor.

Yet, even with surgery the prognosis is often guarded or even poor. The less common pancreatic malignancies, mainly neuroendocrine cancers and lymphomas, have considerably better prognoses.

Several methods are used for detecting and diagnosing pancreatic lesions. Each method has its advantages and disadvantages, including costs, availability, local expertise, operator-dependency, and tradeoffs of accuracy rates for morbidity. Understaging results regarding the resectability of pancreatic tumors leads to undertaking futile and dangerous operations [3,4]. Overstaging of a pancreatic tumor by any of these methods (all of which have some proven fallibility) would lead to a potentially operable lesion being treated with only palliative measures, essentially 'giving up' on rescuing the patient's life, or lead to unnecessary neoadjuvant therapies.

A related issue is that EUS-guided FNA can evaluate gastrointestinal tract submucosal lesions very well, with a near 100% accuracy. A gastrointestinal stromal tumor (GIST) is one of the important histologic/cytologic diagnoses of submucosal lesions [5].

Diagnosing and grading GISTs is of significant importance for further management decisions. Those GISTs staining positively for the c-KIT mutation often respond well to imatinib [6].

There are some controversies regarding the indications for performing EUS-guided FNA. These are several of the key unanswered questions:

- Is it necessary to obtain a tissue diagnosis of a pancreatic mass which appears operable and has malignant features according to a CT scan or EUS?

- Should one order EUS-guided FNA of a CT-inoperable tumor, prior to initiating chemotherapy or radiation therapy?

- Regarding a stable pancreatic cyst, is periodic radiological follow-up sufficient, or is there a need for sampling the lesion for the existence of such indicators as CEA, amylase and lipase.

- When is EUS-guided FNA sampling of a submucosal GI tract lesion indicated? The leading principle in determining an indication for performing any medical test is whether or not the information gathered by this method is likely to change the recommended treatment plans, and thus improve the patient's prognosis.

As access to EUS becomes more widespread, EUS-guided FNA of pancreatic and GI submucosal lesions is becoming increasingly common. In Israel, there has been a rise from fourteen medical centers performing 3700 EUS procedures in 2001 to twenty centers performing 5000 procedures annually, three years later [7].

To date, clear and accepted indications with unison among the relevant physicians regarding when to perform EUS-guided FNA and when to refrain from FNA, are lacking. One landmark study showed that only about 50% of gastroenterologists who treat patients with pancreatic cystic lesions were aware of the existence of such guidelines, and many did not accept the current consensus guidelines [8].

While it presents too small a survey to establish definitive guidelines, the present work humbly aims to formulate a milestone in the final mission of creating a consensus by surveying specialists from the various relevant fields, as to the indications of EUS-guided FNA.

## Methods

### The survey

Based on the areas of controversy mentioned above, a questionnaire was developed by expert consensus with a pilot for validation given by a group of very experienced well-respected specialists in gastroenterology, surgery and oncology. The survey instrument presented five different medical situations for which there is no widely shared consensus.

In the final stage of validation, the questionnaire was handed to a small pilot group of participants in order to confirm that the questions' clarity and that the main idea behind them was well understood.

Following are each of the five specific case-survey questions and summaries of the answers received in the survey.

### Case number 1

A 71-year old patient presents to you with painless jaundice of 2-3 weeks' duration. The patient is a nondrinker, no past hepatitis, CT found a T2N0MO pancreatic head tumor.Would you recommend:

a- EUS, with FNA of the lesion

b- EUS but NO FNA

### Case Number 2

A 68-year old female has a locally advanced T4N0M0 pancreatic tumor as seen on CT scan, inaccessible to CT-FNA, and considered inoperable due to Superior Mesenteric Artery encasement or invasion.

Would you recommend:

a- FNA by EUS.

b- Referral to oncotherapy without cytology.

### Case Number 3

A 60-year old woman with slight dyspepsia was found to have external pressure on the stomach during gastroscopy. CT found a 30 mm multi-loculated cystic lesion of the pancreatic body/tail region.

Would you recommend:

a- EUS- FNA.

b- No FNA- refer patient directly to surgery.

### Case Number 4

A 55-year old patient has a 2.3 cm pancreatic body cyst which has not changed since previous CT exam done 6 months ago. The patient feels fine, serum CA19-9, amylase, CEA and CRP all normal.

Would you recommend:

a- EUS-FNA puncture and drainage of the cyst

b- No FNA

### Case Number 5

A 50 year old patient examined for heartburn is found on gastroscopy to have a 19 mm submucosal lesion of the gastric wall. Before deciding whether to recommend surgery or long-term follow-up, which would you recommend:

a- EUS-FNA of the lesion.

b- No FNA

### Study Participants

Each case focused on in the survey was handed to physicians (by medical student YR at medical conventions or on their wards), who were asked to answer it and return it immediately or send it back by mail. Participants included specialists and residents from three different specialties (table 1): gastroenterology (n = 43), surgery (n = 38) and oncology (n = 20) at four tertiary medical centers and three smaller hospitals. The physicians were requested, as part of the survey, to indicate whether or not they personally treat the relevant patients and how many years of experience they have. Consequently, gastroenterologists were asked whether they perform EUS themselves, surgeons whether they operate on pancreatic lesions themselves and oncologists whether they treat patients with pancreatic lesions and/or GISTs (see tables 2 and 3).

**Table 1:** The Distribution of the Physicians According to Specialties.

Percent (%)	Number	Specialty
19.8	20	Oncologists

42.6	43	Gastroenterologists

37.6	38	Surgeons

100.0	101	Total

**Table 2:** The distribution of Those Physicians Who Treat the Relevant Patients and Those Who Do Not.

	Treat	Do Not Treat	Unknown	Total
Oncologists	35% (7)	35% (7)	30% (6)	100% (20)

Gastroenterologists	16.3% (7)	79% (34)	4.7% (2)	100% (43)

Surgeons	55.25% (21)	34.25% (13)	10.5% (4)	100% (38)

Total	34.6% (35)	53.5% (72)	11.9% (12)	100% (101)

Numbers in parentheses: case number

**Table 3:** The distribution of physicians' experience in years according to specialty.

	0-5	5-10	Over 10	Unknown	Total
Oncologists	40% (8)	5% (1)	25% (5)	30% (6)	100% (20)

Gastroenterologists	30.2% (13)	14.0% (6)	51.2% (22)	4.6% (2)	100% (43)

Surgeons	18.4% (7)	18.4% (7)	60.6% (23)	2.6% (1)	100% (38)

Numbers in parentheses: case number

### Statistics

As recommended by a statistician, a total number of 150 questionnaires were distributed based on a power study suggesting that 100 completed surveys would be sufficient to reach statistically significant results from the intended groups and subgroups. All statistical analyses were performed by using SPSS software. Reported p values were all p < 0.05 as levels of significance.

### Limitations

In this study, an expert consensus, but not a formal validation using internal reliability, was obtained. Each medical case presented could have many minor variables which might change one's decisions regarding EUS-guided FNA. However, the thrust of whether or not to include EUS-guided FNA for the overall situations seems to have been well served by the questions as were finally formulated.

## Results

In total, 101 physicians answered the survey. The distribution of specialties (see table 1) was 43 gastroenterologists, 38 surgeons, and 20 oncologists. Furthermore, the groups were subdivided into two categories: Those who treat the relevant patients by themselves (e.g. a surgeon who performs pancreatic operations, a gastroenterologist who performs EUS, etc.) and those who do not (see table 2). 55% of surgeons, 35% of oncologists, and 16.3% of gastroenterologists personally treat these patients.

### Case number 1

**Figure 1:**
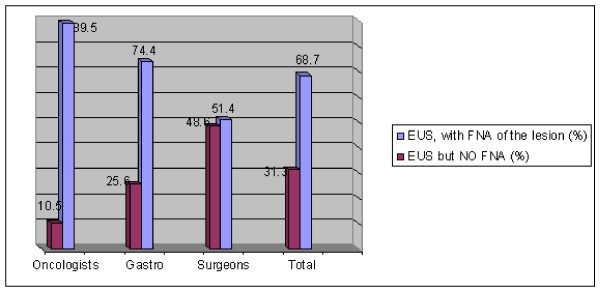
**Case #1: Is FNA recommended for a T2NOMO 2 cm pancreatic lesion?**.

Overall, 68.7% would recommend EUS-FNA of a T2 pancreatic head lesion. There were significant differences between specialties. EUS was considered to be indicated by51% of surgeons, 74.4% of gastroenterologists and 89.5% of oncologists. The statistical differences between oncologists and surgeons is p < 0.01; between gastroenterologists and surgeons, p < 0.05 (See Figure 1).

### Case Number 2

**Figure 2:**
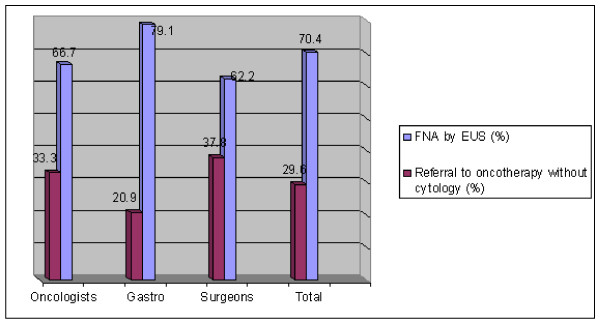
**Case #2: Is EUS-FNA recommended before referral for oncotherapy for a pancreatic tumor encasing the Superior Mesenteric Artery?**.

In total, 70.4 percent of the physicians replied that EUS-guided FNA should be performed in a patient presenting with an inoperable pancreatic tumor characterized to be without metastases according to CT (locally advanced T4N0M0). The breakdown by specialty, of those writing that FNA was indicated, was 62% of surgeons, 67% of oncologists, and 79.1% of gastroenterologists (See Figure 2).

### Case Number 3

**Figure 3:**
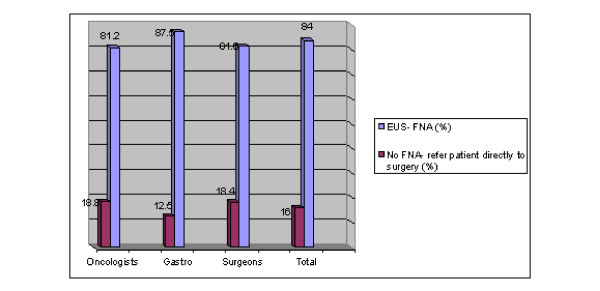
**Case #3: Would you recommend EUS only or EUS- with FNA for a three-cm pancreatic cyst/cystic lesion?**.

The overall mean response was that 84% wrote that FNA is indicated before deciding on surgery for a symptomatic 30 mm multi-loculated cystic lesion of the pancreatic body/tail region. The group breakdown was relatively homogeneous: 81.6% surgeons 81.2% of oncologists and 87.5% of gastroenterologists (See Figure 3).

### Case Number 4

**Figure 4:**
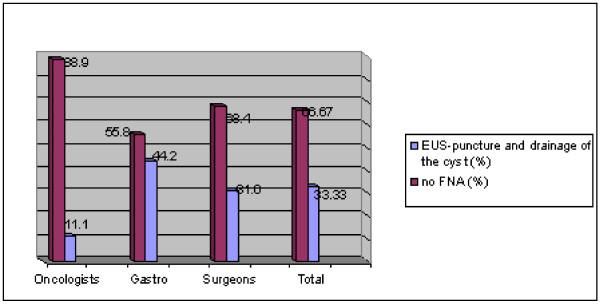
**Case #4: Would you recommend EUS-FNA for a stable 23 mm pancreatic cyst seen to be stable and asymptomatic for 6 months by repeated CT scans?**.

In the case of an apparently 23 mm stable asymptomatic pancreatic cyst with normal serum markers, 66.67% of physicians support non-invasive management. By specialty, FNA was written as indicated by 31% of surgeons, 11% of oncologists, and 44.2% of gastroenterologists. Statistical differences were found between oncologists and gastroenterologists at p < 0.05 (See Figure 4).

Of those gastroenterologists performing EUS by themselves, 71.4 percent (n = 5) opted for EUS-guided FNA of the lesion, whereas, 61.7 percent (n = 21) of those who do not perform EUS by themselves, wrote that conservative measures are preferable (p = NS).

### Case Number 5

**Figure 5:**
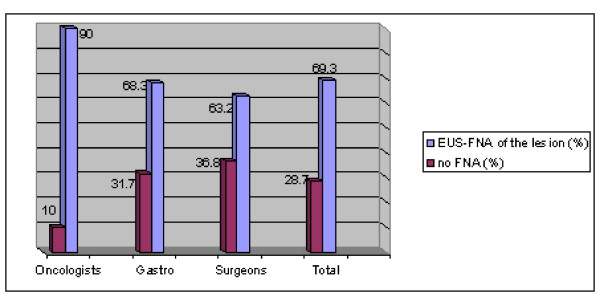
**Case #5: For a 19 mm submucosal lesion of the gastric wall - Would you recommend performing EUS-FNA of the lesion or not?**.

More oncologists (90 percent), as opposed to only 63.2 to 68.3 percent of the other groups wrote that cytological evaluation by EUS-FNA is indicated for a 19 mm gastric submucosal lesion prior to deciding on surgery. The statistical difference between oncologists and surgeons is p < 0.05 (See Figure 5).

## Discussion

Prudent applications of the many expensive and sometimes time-consuming modalities which may assess pancreatic lesions, continues to challenge clinicians. Local expertise and availability and costs are all parameters which may impact on decisions for staging before resections of these lesions.

Worldwide, some medical centers may inform patients that they had benign not malignant disease (such as autoimmune or focal pancreatitis) only after undergoing a futile major operation. In Israel, which has 18 EUS centers for a population of 7.5 million persons, such futile surgery is widely considered to be unacceptable. Conversely, negative FNA could result in delaying surgery for potentially resectable tumors. As demonstrated in the first case, for T2N0MO pancreatic lesions, in contrast to gastroenterologist and oncologist opinions (73.3-89.5%), only 51.4 percent of surgeons wrote that FNA is indicated prior to surgery. This is one of the most contentious issues dividing physicians. While a positive FNA confirms need for surgery, negative FNA does not necessarily refute the need for surgery. The frequency which one accepts of finding post-hoc that surgery was performed only to find a focal pancreatitis of any cause is at the focus of the debate. In Israel, every effort to prevent unnecessary surgery generally includes attempting FNA and having cytological confirmation if possible. In places where FNA is unavailable, it might seem much more acceptable to attempt surgery based on imaging alone. The false positive rate of surgery may be compared to the rate of operating on what turn out to be "white or non-inflamed appendix". In young male patients, the negative appendectomy rate should be low, perhaps under10%; this means that less than 10% of misdiagnoses proven at surgery are considered an indication that too many true appendicitis cases are likely being missed. The morbidity and mortality risks of pancreatic surgery are considerably higher than for appendectomy. Also, the urgency for operation of the pancreas is less than for appendectomy, for which a delay in hours could prove disastrous. Thus, the reluctance to accept unnecessary operations may arguably be lower for pancreatic operations, and the thoroughness of establishing need for surgery for pancreatic lesions should be maximal.

Most physicians in this survey (68.7%) wrote that it is recommended to perform EUS-guided FNA to evaluate an EUS demonstrated pancreatic lesion prior to surgical intervention. It is interesting to consider why surgeons support this measure to a lesser extent in comparison to their peers (only 51.4% of surgeons). The very physicians most at risk of disappointing patients were found to be most likely to decide on operations which might be futile. The threshold of data needed to decide to operate by surgeons was found to be lower than among others.

In the second case, it appears that most physicians believe that cytology should be attempted prior to commencing chemotherapy in a patient with an inoperable pancreatic tumor according to CT. Supporting this decision are two key considerations: 1- that cytological diagnosis could determine the specific tumor type and thus change the proper choice of chemotherapy in 8-10% of cases which are not primary pancreatic adenocarcinomas and 2- to avoid treating with chemotherapy a benign inflammatory lesion of the pancreas. Once again, the consideration of how heavy a preponderance of evidence indicates a need for a specific therapy (in this case chemotherapy) involves the tendency of physicians to make risky decisions which could potentially be avoided by cytology. The risk rate of patients receiving chemotherapy wrongly treated for a benign condition/misdiagnosis has not been reported specifically for pancreatic lesions. The risks of the specific chemotherapy being offered vs. the difficulties involved in getting cytological or histological confirmation are part of an equation involving clinical cultural and legal environments.

The third case reveals that most physicians, regardless to their specialty, believe that a symptomatic pancreatic cyst, even if smaller than 30 mm, should be aspirated rather than referred directly for surgery. Considerations supporting EUS-FNA include the possibilities raised by results of testing the cystic fluid for CEA, amylase and cytologically determining the presence of glycogen-rich cells- all which would raise the accuracy of evaluating the risk of malignancy. Pseudocysts and serous cystadenomas are cases for which a watch and wait strategy would dominate, as opposed to premalignant or malignant cysts which indicate need for require surgical intervention.

In the fourth case described, the majority of physicians answering the survey indicated that the management of an asymptomatic and stable pancreatic cyst, smaller than 30 mm, is to watch and wait, meaning, follow-up only. Some disagreement was found within the sub-groups of gastroenterologists. Of those performing EUS by themselves, most believe that the cyst should be drained, whereas, most of those who do not perform EUS by themselves, recommended more conservative management. This controversy appears to exist between the ASGE and ACG guidelines. The former, emphasizing the risk that every cystic lesion of the pancreas, regardless of its size, may be malignant (or premalignant) recommends diagnostic evaluation and mentions the clinical useful information added by assessing the cystic fluid for tumor markers, amylase and lipase. The latter, however, allows cross sectional imaging follow-up as long as the lesion is smaller than 5 mm and remains asymptomatic and not growing. Increasingly, a cyst which has all of the EUS features of a serous cystadenoma is considered a low risk lesion, and deferring FNA and making the diagnosis based on EUS imaging alone is considered an option [9,10].

According to this survey most physicians would avoid the risks of FNA. This approach, compared to endosonographers, may be due to concerns about the risks, unawareness of the benefits, and/or considerations of availability and costs of EUS-FNA. The risks of FNA, have recently been estimated in a large meta-analysis. Mortality was found to be about 1/5000, while significant morbidities including post-FNA pain and post-FNA pancreatitis were more common, especially among the prospective studies [11].

As evident by the fifth and final case, approximately 70 percent of physicians wrote that cytological evidence should be sought for a suspected GIST. Modern immunohistochemistry tests for c-KIT and PDGFRA can diagnose GIST. Impacts of FNA in such a situation are that low-grade small GISTs may require no therapy, whereas high-grade GISTs respond very well to imatinib treatment. It is crucial to note that there are many other intramural/submucosal lesions which could be diagnosed by EUS- FNA in this situation, some of which would have therapeutic impact (e.g. carcinoid).

A final caveat is that FNA is far from a perfect diagnostic modality. The ability to obtain accurate samples from EUS-guided FNA varies from 50 to about 90%, depending on the organ being sampled, the expertise, and possibly the presence of a cytologist during the procedure [12]. Even for cases of thyroid nodules, for which FNA is done from outside of the body, under relatively direct vision, and being able to regulate all body part movements which could adversely affect the procedure and lead to false negative due to sample error there is a significant (roughly 20%) rate of non-informative cytology results [13].

In the case of a pancreatic lesion visualized by CT, performing EUS-guided FNA prior to surgical or oncological treatment may provide (in 85% of cases) supporting cytological evidence. The role of cytology in a case in which, according to the CT, the pancreatic tumor is inoperable, (e.g. due to vascular involvement), is to determine the proper chemotherapy/radiotherapy, especially for atypical tumors.

The symptoms associated with pancreatic cysts are hard to establish. A pancreatic cyst in a symptomatic patient is an indication for cytological evaluation, and/or for drainage, with different methods being available depending on cyst etiology, size, and other variables [14,15]. The search for more dependable cyst fluid biomarkers than CEA continues: most recently MUC7 was found to have significant value in determining likelihood of a need for surgery and malignant potential of cysts [16].

Conservative measures in an asymptomatic patient presenting with a small and stable cyst, according to radiography, is widely considered to be sufficient.

A gastric submucosal lesion may be a GIST, a carcinoid or a benign harmless lesion, and so it should be aspirated in order to determine its stage and the treatment. EUS-FNA has become an essential tool in the diagnosis and staging of pancreatic tumors. The major advantage of EUS-FNA lies in the ability of EUS to detect an unresectable disease, to prevent unnecessary surgical exploration and to diagnose small lesions undetectable by other imaging modalities. EUS-FNA can be both therapeutic and diagnostic. The diagnostic values of the FNA are many - these have subjective value. As Brugge, and others since, have summarized, "The chief advantage of EUS-guided FNA is the ability to target, small, intra-pancreatic masses" [17]. Most recently, EUS-guided interventions using FNA needles are being attempted. Novel EUS based techniques are emerging as reasonably safe minimally invasive alternative to the surgical or radiological approaches [18]. As FNA comes into increasing utilization, the measurable impacts in these various real-life situations can be better established.

Most recently, the European Society for Gastrointestinal Endoscopy published its guidelines for EUS-FNA [19]. These guidelines take into consideration the many hundreds of developments which have been discovered and published in the five years since the International Society of Pancreatologists (ISP) reported their consensus guidelines [20]. The ISP guidelines, as mentioned above, went largely unnoticed by the majority of endosonographers, at least insofar as reported in a major survey by Buscaglia et al in 2009 [8]. The present study differs significantly from the ESGE guideline, in that the ESGE guidelines are written only by endoscopists/gastroenterologists, whilst the present work presents the differences in perspectives of surgeons, oncologists and gastroenterologists. Bringing the perspectives of the referring physicians and end-users of the "products" of EUS-FNA may help elucidate the professional needs of the multidisciplinary team who treat these patients.

## Conclusions

Cooperation between gastroenterologists, surgeons and oncologists is essential in order to optimize patients' therapy. This cooperation may lead to better utilization of the various resources and, with no less importance, may also prevent unnecessary procedures, inappropriate administration of chemotherapy and operations and hospital admission. Physicians may tend to emphasize different risks and benefits to patients based on personal experience in different subspecialties of medicine. Thus, the oncologist may be most wary of giving chemotherapy without prior establishment of cytological or histological gold-standard proof of a diagnosis. Surgeons may attempt risky operations on patients if they have maximally solid proof that the operation is indicated. The frequency of surgery for autoimmune pancreatitis (mistakenly misdiagnosed as being a tumor) is not a rare occurrence, and it is one which all involved physicians aim to minimize. The risks of FNA and the risk to benefit ratio are two considerations which gastroenterologists weigh perhaps most carefully when considering how diagnosis will impact management.

Collaborative meetings should be held to determine management. Local availability of expertise and facilities, and local experience, should help narrow the differences in expectations amongst the specialists in teams handling pancreatic tumors and GISTS. This data should reach not only those specialists surveyed in the present study which involved oncologists, surgeons and gastroenterologists, but also should also be available to the primary care physicians who should ideally be involved and coordinate the patients' care and treatment. Careful consideration of the needs and concerns of everyone in the multidisciplinary professional team treating these patients is emphasized so that we may all best serve the patients with these lesions.

## Competing interests

The authors declare that they have no competing interests, including financial and non-financial. This study was not funded, but was rather the focus of Yoav Rosenthal's MD thesis.

## Authors' contributions

JL was the initiator and PI of the study. YR Was active in every phase of this study, from planning to write-up, which was performed in compliance with the requirements of the Technion for an M.D. thesis. Finally, YK shared in developing the questions in the survey, particularly for surgical aspects, and supported the distribution and collection of surveys. All authors read and approved the final manuscript.

## Pre-publication history

The pre-publication history for this paper can be accessed here:

http://www.biomedcentral.com/1471-230X/11/117/prepub
